# Electrochemical Corrosion Behaviour of Different Grades of WC-Co, High-Cr White Cast Irons and Hadfield Steel in 1 M Sulphuric Acid

**DOI:** 10.3390/ma14206130

**Published:** 2021-10-15

**Authors:** Johannes H. Potgieter, David Whitefield, Vivian Motsumi

**Affiliations:** 1 School of Chemical and Metallurgical Engineering, University of the Witwatersrand, Private Bag X3, P.O. Wits, Johannesburg 2050, South Africa; Herman.potgieter@wits.ac.za (J.H.P.); 670537@students.wits.ac.za (V.M.); 2 Department of Natural Sciences, Manchester Metropolitan University, Chester Street, Manchester M1 5GD, UK

**Keywords:** WC-Co alloys, hadfield steel, High-Cr white cast irons (HCWCI), mining industry, tribocorrosion

## Abstract

Electrochemical polarisation tests were carried out on three grades of WC-Co cemented carbides to investigate the corrosive behaviour of the hardmetals and rank them as viable protective liners for chutes and skips in the mining industry. The cobalt binder content and WC particle size varied. The binder content ranged from 6–12 wt%, and the grain size of the WC particles ranged from 0.4–2.3 µm. The performance of the WC-Co hardmetal was compared to three different grades of high chromium white cast irons and Hadfield steel. The cast irons varied in both their chromium content and the morphology of the Cr-rich primary carbides. Potentiodynamic polarisation and linear polarization resistance scans were used to determine the corrosion current density and other electrochemical parameters. The microstructural characteristics of the samples were analysed using Scanning Electron Microscope(SEM) with Energy Dispersive Spectroscopy (EDS), and optical microscopy. The potentiodynamic scans revealed that, although the WC-Co alloys were found to have generally improved corrosion resistance, it was the high-Cr white cast iron (22 wt% Cr) that recorded the lowest corrosion current density and therefore displayed the best resistance against corrosive attack in 1 M H_2_SO_4_. The Hadfield steel exhibited the poorest resistance to corrosion and therefore, suffered the most degradation to its exposed surface.

## 1. Introduction

The movement and storage of ore in the mining and minerals processing industry requires chutes and skips. Large structural items in the mining industry like chutes and skips consume large quantities of engineering materials [1]. In order to prevent production stoppages and a possible substantial loss of capital due to the premature mechanical failure of these structural components, there is a need to design wear- and corrosion-resistant liners at specific locations of high deterioration to limit the damage experienced during operation [2,3,4,5,6,7,8,9,10,11,12,13,14]. Corrosion wear is just as severe on ore transportation structures as mechanical wear, which is why it is wise to also rank the corrosive response of a material when selecting a protective liner for chutes and skips.

There are many wear and corrosion mechanisms involved during the handling of ore in mines [1,11,12]. Understanding the synergistic effects of these surface-degrading mechanisms enables the engineer to design and develop lining plates or similar surface-protective methods that would offer optimal effectiveness in protecting the components against both wear and corrosion [2,15]. Since chutes and skips are not only subjected to mechanical wear in mining applications but are also in contact with chemically aggressive environments, the selection of the lining materials should also be designed for ultimate corrosion resistance. This present study is aimed at evaluating the electrochemical responses of different grades of tungsten carbide cobalt (WC-Co), high chromium white cast irons (HCWCI) and Hadfield steel, and determining which material offers the best resistance against corrosive attack in 1 M H_2_SO_4_.

WC-Co cermets are hard monocarbide grains cemented in a soft binder matrix of tough cobalt metal by liquid phase sintering [16]. Although WC-Co hardmetals are known for their excellent resistance to wear [17,18,19,20], they have also demonstrated fair resistance to chemical attack in corrosive environments [21,22,23,24]. Generally, the two-phased WC-Co cermets do not passivate in acidic media [25,26]. The process of corrosion in aggressive media favours the selective dissolution of cobalt, while the WC particles remain inert, leaving a skeleton of WC particles on the surface [21]. The WC particles of the hardmetals can also corrode, but this only occurs at significantly higher potentials [27]. The process of corrosion of WC-Co hardmetals is shown in Equations (1) and (2) [28].

Co → Co^2+^ + 2e^−^ (1)

WC + 5H_2_O → WO_3_ + CO_2_ + 10H^+^ + 10e^−^ (2)

In their studies, Human and Exnar [18] investigated other properties of WC-Co hardmetals that could possibly influence their resistance to corrosion, and it was found that grain size had no effect on the electrochemical attack of the hardmetals in 1M H_2_SO_4_. However, recent research shows that the microstructural characteristics such as growth inhibitors and the grain size of WC particles influenced the electrochemical corrosion resistance of the WC-Co cemented carbides [29,30,31,32]. Liu et al. [29] found that there is a linear relationship between the grain sizes and the corrosion current density. The chemical nature of the matrix, such as the addition of grain growth inhibitors to ultrafine-sized powders, has also been found to significantly influence the corrosion behaviour of WC-Co cemented carbides [33]. Another general trend observed was that the corrosion rate of WC-Co alloys increased with an increase in Co content. Human and Exner [18] found that the composition of the binder phase was of great importance to the corrosion resistance of cemented carbides They added that the alloys experienced a mass loss during corrosion when all other parameters, such as the grain size of the WC particles, were kept constant. Similar to the WC-Co cemented carbides, HCWCI are not only exceptional at resisting severe abrasive and erosive environments, but they have also proven to perform well in different corrosive environments. Their resistance to chemically corrosive environments, such as reducing acids, has been found to be strongly influenced by the presence of high levels of alloying elements [34]. Tian et al. [35] and El-Aziz et al. [36] reported that alloys that contained high ratios of Cr/C in their microstructures were the most suitable to resisting damage by corrosion. This implies that the carbon content has to be relatively low so that excess concentrations of chromium can be found in the matrix to further improve the corrosion resistance of the matrix [34,37]. Furthermore, El-Aziz et al. [36] discovered in their work that a high-volume fraction of large bulky primary carbides in the form M_7_C_3_ were advantageous in harsh corrosive environments by forming secondary carbides and primary eutectic carbides.

High-manganese austenitic steels, also known as Hadfield steels (HS), are traditionally used as wear-resistant alloys in harsh applications wherein high impact abrasion, high fracture toughness and moderate resistance to corrosion are required [38,39]. The corrosion of conventional steels in acidic media is one of the most common failure mechanism in the minerals processing industry [40]. There are a few studies available on the corrosion behaviour of Hadfield steels in acidic media [40,41,42,43]. In their studies, Grajcar et al. [40] reported that the substantial mass loss of high-manganese steel in 1 M H_2_SO_4_ solution was due to the hydrogen depolarisation mechanism, which was subsequently accompanied by the local cracking of the previously formed product layer, resulting in the protective passive layer being removed during the polarisation process, causing further significant damage to the exposed and unprotected surface [42,44,45]. Moreover, MnS inclusions were reported to act as initiating sites of pitting corrosion in steels [41].

To study the comparative corrosion resistance of the investigated materials, potentiodynamic polarisation scans were carried out. The surfaces of the corroded samples were examined and characterised using Scanning Electron Microscope- Energy Dispersive Spectroscopy (SEM, EDS), and light optical microscopy. The investigated metals were exposed to an acidic electrolyte (H_2_SO_4_) because most chutes and skips are exposed to acid-ironstone/acidic underground water in the mining and mineral processing industry. Furthermore, this medium provided a fast and easy way to compare the corrosion resistance of the different alloys against each other.

This investigation had two main foci, namely, firstly to compare the corrosion resistance of the typically employed WC-Co alloys with the cheaper Hadfield steel widely applied in the mining industry and secondly to establish whether both the WC-Co alloy group and the Hadfield steel could be replaced by much cheaper, and at this stage, experimental high-chromium white cast iron compositions. The successful achievement of the latter goal would offer the mining industry real economic advantages if acceptable and comparable corrosion resistance compared to the Hadfield steel and the WC-Co alloys could be achieved. At the same time, the production of experimental high-chromium white cast irons as production materials could create new uses for chromium in the mining industry and lead to increased job creation opportunities in an economy with unacceptably high unemployment rates.

## 2. Experimental Procedure

### 2.1. Metallographic Preparation

The as-received samples were sectioned into 10 mm × 10 mm × 5 mm blocks using a precision cutting machine. The samples were cleaned with ethanol after cutting to remove any debris formed during cutting. The test surfaces of the samples were wet-ground using silicon carbide papers with sizes ranging from 220- to 1200-grit to achieve a flat top surface that was free from coarse scratches. Thereafter, a mirror-like surface was achieved with a 3 µm pan cloth followed by a 1 µm diamond spray. The specimens were washed with distilled water and the polished surfaces of the WC-Co and Hadfield steel samples were etched using Murakami’s reagent and 5% Nital, respectively. The HCWCI samples required no etching since the microstructures of the alloys were easily revealed during polishing using colloidal silica. The tested area for all samples was kept at 0.25 cm^2^. The chemical composition of the samples used in this study, viz. WC-Co, HCWCI, and Hadfield steel, are summarised in Table 1 and Table 2.

### 2.2. Microstructure Analysis

An optical microscope and scanning electron microscope (SEM){“Scanning electron microscope:(SEM)”} were used to examine the microstructures, surface morphology and chemical analysis of the investigated samples in their as-received condition and after the electrochemical tests. The optical microscope used was an Olympus SC50 The SEM used was the Zeiss high-vacuum scanning electron microscope (Zeiss, Jena, Germany) equipped with an energy-dispersive X-ray spectrometry (EDS){“Energy dispersive x-ray spectrometry:(EDS)”}. The SEM-EDS operated at 20 keV and the micrographs were taken in both backscattered electron (BSE){“Backscattered electron:(BSE)”} and secondary electron (SE){“Secondary electron:(SE)”} mode. ImageJ software (Zeiss, Jena, Germany) was used to determine the grain sizes of the investigated specimens.

### 2.3. Potentiodynamic Corrosion Testing Procedure

A 1 M H_2_SO_4_ solution was used for the corrosion testing. A fresh solution was prepared for each test. A cylindrical three-electrode 900 mL Pyrex glass cell was used for this experiment. The cell consisted of a graphite counter electrode, a silver/silver chloride reference electrode immersed in a 3 M KCl solution and the working electrode. The potentiodynamic polarization scans were carried out at room temperature (25 ± 1 °C) in a water bath where the temperature could be closely regulated. The electrodes were connected to an Autolab potentiostat (Metrohm, Herisau, Switzerland) and the characteristic electrochemical parameters such as the corrosion potential (E_Corr_), current density (i_Corr_) and the corrosion rates (CR) were defined by using Nova software. The test procedure was set as follows:

1. Open circuit potential (OCP){XE “Open circuit potential:(OCP)”}for 1 h.
2. Linear potentiodynamic scans.

The scans were carried out from −0.2 V versus the OCP to 1.5 V versus the reference electrode. The scan rate and step potential were set at 5 mV/s and 1.6 mV, respectively. Each experiment was conducted in triplicate to ensure the repeatability of the results.

## 3. Results

### 3.1. Metallurgical Microstructure of the Investigated Samples

The grain sizes of the investigated specimens are summarized in Table 3. Because the morphology of the primary carbides found in the HCWCI alloys are mostly continuous, their sizes were determined based on 80% of their area range per analysed alloy.

#### 3.1.1. WC-Co Cemented Carbides

**Figure 1:**
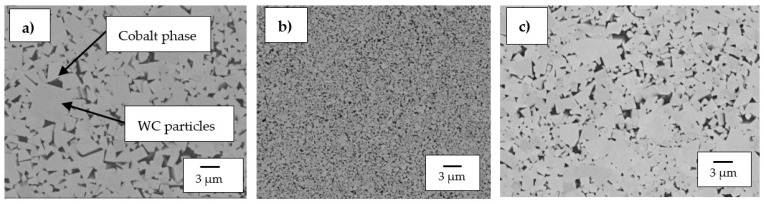
SEM micrographs of the investigated WC-Co alloys: (**a**) WC-8Co, (**b**) WC-12Co, and (**c**) WC-6Co.

Figure 1a–c are the SEM micrographs of WC-8Co, WC-12Co and WC-6Co, respectively. The binder content (Co) of the cemented carbide samples ranged between 6–12 wt.%, and the samples had grain sizes between 0.4–2.3 µm. WC-12Co contained the finest WC grains in its structure, whereas WC-6Co exhibited a much coarser structure. The refined structure of WC-12Co can be attributed to the chromium present in the form Cr_2_C_3_.

#### 3.1.2. High-Cr White Cast Irons

**Figure 2:**
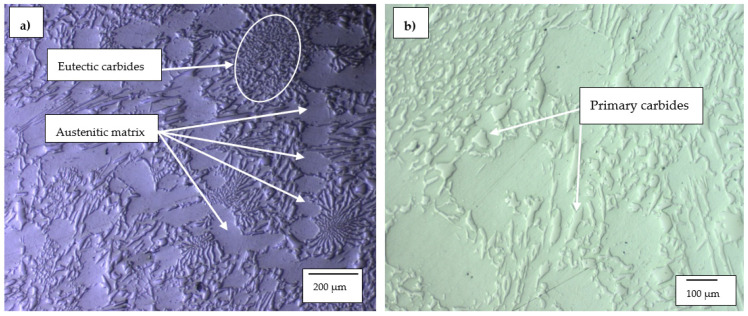
Optical microscope micrographs of the investigated high-chromium white cast iron alloy: (**a**) HCWCI-1 at 5× and (**b**) HCWCI-1 at 20×.

**Figure 3:**
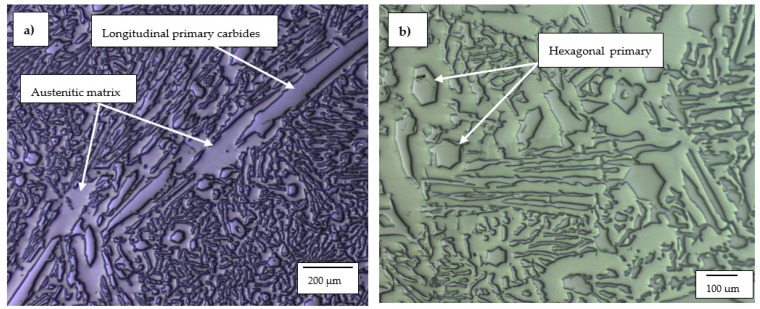
Optical microscope micrographs of the investigated high-chromium white cast iron alloy: (**a**) HCWCI-2 at 5× and (**b**) HCWCI-2 at 20×.

**Figure 4:**
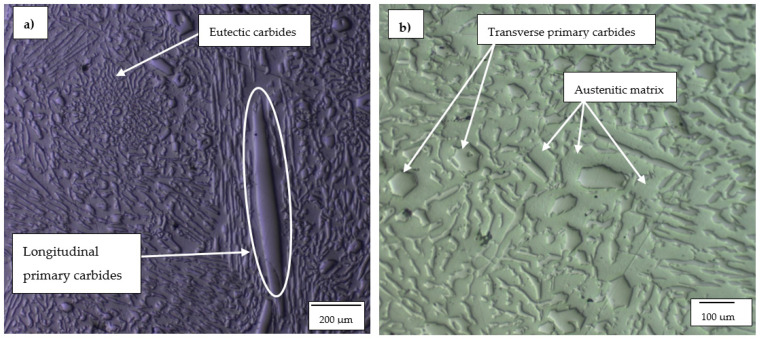
Optical microscope micrographs of the investigated high-chromium white cast iron alloy: (**a**) HCWCI-3 at 5× and (**b**) HCWCI-3 at 20×.

The optical micrographs of HCWCI-1, HCWCI-2 and HCWCI-3 are shown in Figure 2, Figure 3 and Figure 4, respectively. HCWCI-2 and HCWCI-3 exhibited larger primary carbides in their microstructures than HCWCI-1. This is because of their increased Cr contents, which promote the formation of Cr-rich carbides. HCWCI-3 exhibited larger longitudinal primary carbides and larger hexagonally shaped transverse primary carbides. This is because Cr stabilizes carbides during the solidification of white cast irons, hence promoting the formation and dispersion of hard Cr-rich and (Fe, Cr_x_)C_Y_ carbides. All three alloys contained a high density of rod-shaped and isolated M_7_C_3_ carbides, which are expected when the Cr content exceeds 15% [46,47]. The ImageJ software revealed that about 80% of the primary carbides of the HCWCI-1 had an area in the range 25–1450 µm^2^, whereas the primary carbides of the HCWCI-2 and HCWCI-3 alloys had an area in the range 280–5330 and 65–2745 µm^2^, respectively.

#### 3.1.3. Hadfield Steel

**Figure 5:**
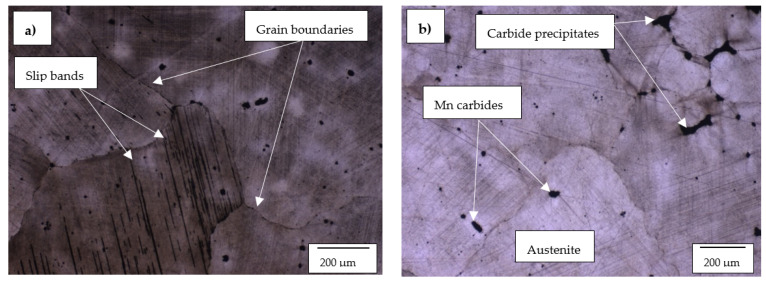
Optical microscope micrographs of the investigated Hadfield steel showing (**a**)the grain boundaries, slip bands and (**b**) carbide precipitates.

The optical micrographs of the Hadfield steel alloy are shown in Figure 5a,b. The Mn and C composition of the investigated Hadfield steel are 12.4% and 1.1%, respectively. This implies the steel’s Mn/C composition ratio lies within the original standards of the austenitic manganese steel. The Hadfield steel consists of a Cr content of 0.3 wt%, which is essential for the yield strength and corrosion resistance of the Hadfield steel [48,49]. The Hadfield steel had a grain size of 300 µm.

### 3.2. Electrochemical Corrosion Behaviour of the Investigated Alloys

#### 3.2.1. Open Circuit Potential (OCP)

**Figure 6:**
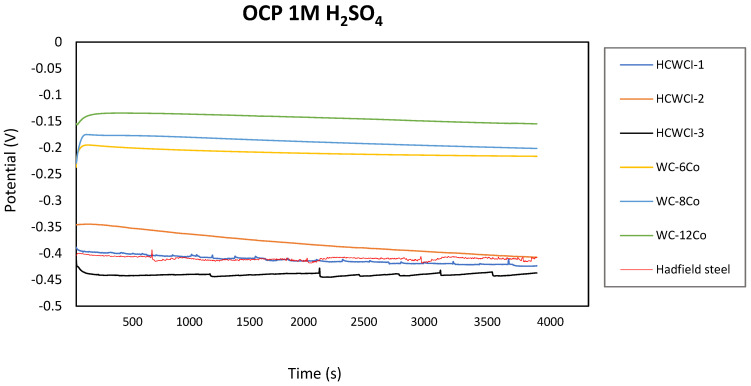
Open Circuit Potential behaviour of the investigated metals in 1 M H_2_SO_4_.

The OCP (E_OC_) values and the variations therein were measured for an hour in 1 M H_2_SO_4_ as presented in Figure 6. The recorded OCP values started off unstable, displaying random fluctuations in the first few minutes of measuring. The Hadfield steel was observed to be the alloy showing less stability, followed by HCWCI-3 and then HCWCI-1. WC-12Co displayed the highest starting potential (most noble) and finally stabilised at a higher potential than the other investigated carbide metals. The HCWCI-3 alloy was observed to have displayed the lowest starting potential (least noble) and continued to stabilise at the lowest potential when compared to the other metals. The WC-Co alloys behaved similar as a group and displayed similar trends during the measuring of the OCP. This trend was similar for all the iron-based alloys (HCWCI alloys and the Hadfield steel).

#### 3.2.2. Potentiodynamic Polarisation Measurements

**Figure 7:**
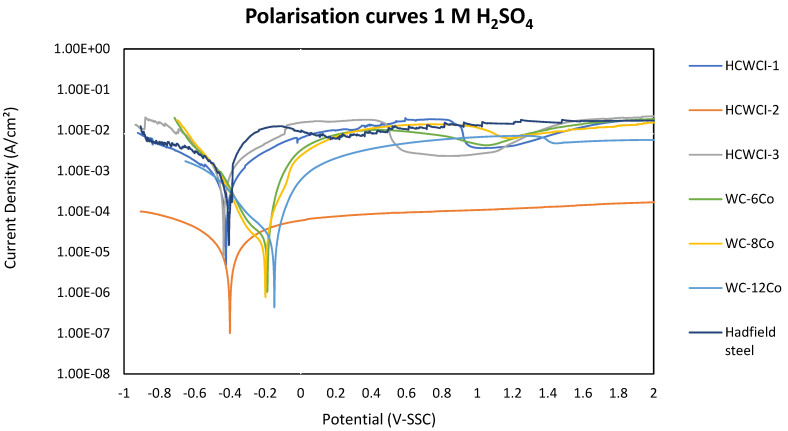
Potentiodynamic polarisation curves of the WC-Co alloys, HCWCI alloys and Hadfield in 1 M H_2_SO_4_.

The polarisation scan curves are presented in Figure 7. The curve profiling of the samples showed a typical active-pseudopassive transition behaviour (although showing small passive regions) with the exception of HCWCI-2, which did not show any passive behaviour. Again, the WC-Co curves showed similar behaviour and the iron-based metals gave similar curves. Comparing the potentials of WC-6Co and WC-8Co, WC-6Co displayed a lower tendency to corrode. This was expected because WC-6Co contained a smaller amount of cobalt in its system, which directly influences the corrosion of WC-Co cemented carbides [18].

Table 4 gives a summary of the electrochemical parameters of the WC-Co, HCWCI and Hadfield steel alloys obtained from the electrochemical polarisation scans using 1 M H_2_SO_4_ as a corroding medium.

The corrosion potential values (E_Corr_) {XE “Corrosion potential values:(E_Corr_)”} of the WC-Co alloys became less noble with an increase of cobalt, with the exception of WC-12Co, which contained the highest Co content but exhibited the most noble value of −0.149 V. In the same manner, WC-12Co also exhibited the lowest value for current density (185 µm/cm^2^) and suffered minimal corrosive attack compared to WC-8Co and WC-6Co, which recorded current density values of 359 µm/cm^2^ and 399 µm/cm^2^, respectively. The i_corr_ value of WC-6Co was very similar to the i_corr_ exhibited by WC-8Co, which was expected considering that the two alloys had similar WC particle sizes, lower Co content and no other alloying elements. Generally, WC-Co cemented carbides with low Co contents have lower i_corr_ values when compared with WC-Co alloys with higher Co [18,50]. However, WC-12Co exhibited the lowest i_corr_ value and thus, exhibited the lowest corrosion rate (CR) value of the three investigated WC-Co alloys.

Although there was little difference between the E_Corr_ values of the HCWCI alloys, HCWCI-2 showed the most noble E_Corr_ value (−0.401 V), while HCWCI-1 and HCWCI-3 gave E_Corr_ values of −0.423 and −0.437 V, respectively. An increase in Cr was advantageous to HCWCI-2 and detrimental to HCWCI-3. This trend was observed with the alloys’ i_corr_ and CR values. An increase of Cr from 20 wt% (HCWCI-1) to 22 wt% (HCWCI-2) had a tremendous effect on the i_corr_ and CR value as both values decreased by an order of magnitude of three and two, respectively. A further increase of Cr to 25 wt% (HCWCI-3) had an adverse effect on the alloy’s resistance to corrosion. HCWCI-3 experienced slightly higher corrosion resistance than HCWCI-1. This influence of Cr on the resistance of the high-chromium cast iron to corrosion was also observed by other researchers [51].

Hadfield steel exhibited the poorest resistance to corrosion compared to the other investigated metals but exhibited an E_Corr_ value (−0.406 V) that was similar to that displayed by the cast iron alloys. The i_corr_ and CR values of the Hadfield steel were very similar to those of HCWCI-1.

Of the WC-Co alloys, it was the WC-12Co sample that showed the best resistance to corrosion based on its recorded i_corr_ and therefore, CR values during the potentiodynamic and linear polarization resistance scanning. Since WC-12Co contains the highest content of the binder phase, one would expect WC-12Co to exhibit the highest tendency to corrode when compared to the other two WC-Co alloys. However, the alloy has a very fine microstructure that consists of WC particles that are very closely packed together. This kind of structure prevents the binder phase from being exposed to the corroding medium, hence preventing excessive corrosion. Additionally, the alloy also has traces of Cr, which has been proven to be beneficial to the WC-Co’s ability to resist corrosion under acidic conditions [52].

The surface of the WC-12Co suffered little to no chemical damage after electrochemical polarization tests. WC-6Co and WC-8Co experienced similar corrosion rates, and therefore, they exhibited the same degree of damage on their surfaces. The corrosion behaviour of the investigated WC-Co alloys was noticed to be strongly influenced by the grain size and morphology of the WC particles. This is in contrast to the studies recorded by Human and Exner [18]. They found that the grain size and morphology of WC-Co hardmetals had no significant effect on the carbides’ resistance to corrosion in an acidic environment.

Of all of the investigated specimens, HCWCI-2 exhibited the best overall performance resisting corrosion. The alloy displayed negligible damage when exposed to an acidic environment. The chromium content and the morphology of the Cr-rich carbides was shown to be crucial for corrosion resistance in white cast irons and therefore contributed to its exceptional corrosion resistance.

Hadfield steels responded the worst during electrochemical tests and displayed the most damage to its exposed surface.

The tendency of these investigated alloys to corrode can also be attributed to their reinforcing particles, viz. WC in the WC-Co hardmetals and the Cr-rich primary carbides in the white cast irons. In a study investigating the corrosion behaviour of WC and Cr_3_C_2_-based coatings by Luiz et al. [53], the Cr_3_C_2_-based coatings outperformed the WC-based coatings in resisting corrosive attack. This was seen to also be the case between the two best-performing alloys (WC-12Co and HCWCI-2) investigated in this study. The Cr_3_C-based alloys, such as the Cr-rich primary found in HCWCI-2, proved to be more inert to chemical degradation in 1M H_2_SO_4_ than WC particles.

### 3.3. Analysis of the Microstructure after Electrochemical Test

#### 3.3.1. WC-Co Cemented Carbides

**Figure 8:**
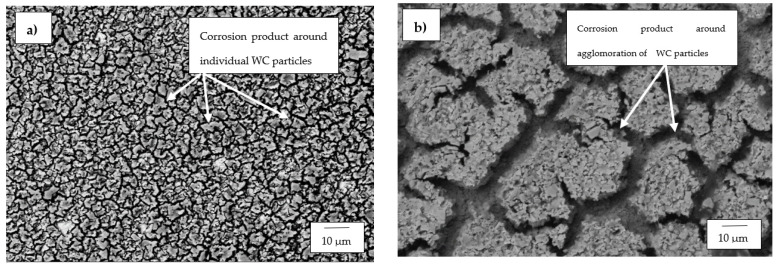

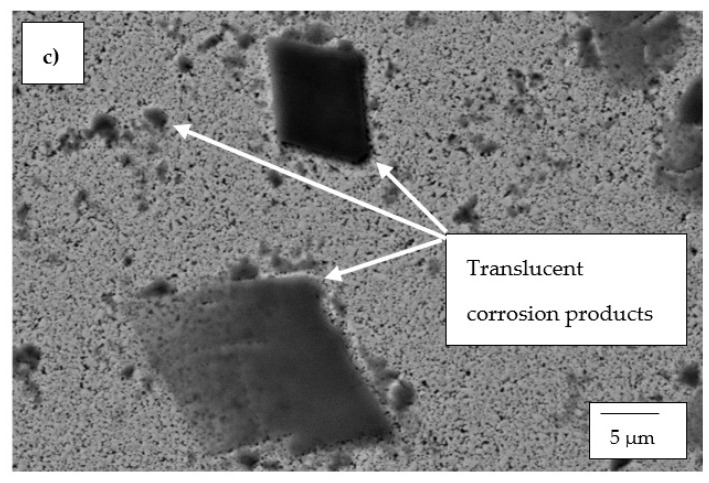
SEM micrographs of: (**a**) WC-6Co, (**b**) WC-8Co and (**c**) WC-12Co after undergoing electrochemical tests in 1 M H_2_SO_4_.

**Figure 9:**
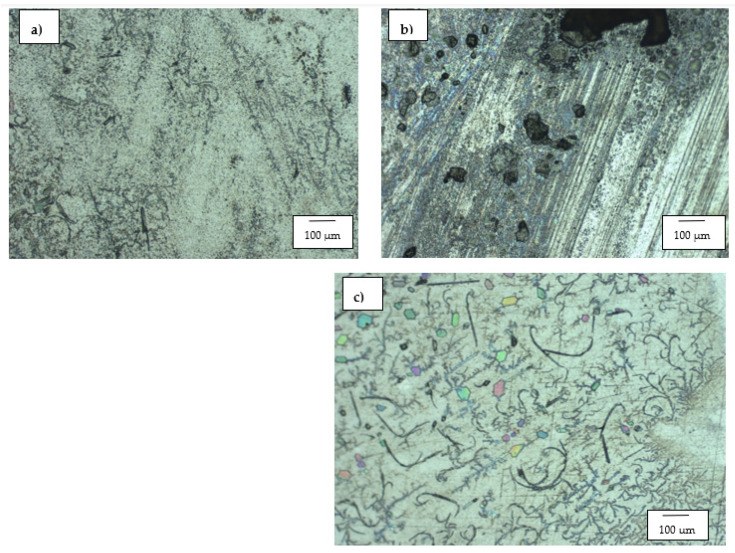
Optical micrographs of: (**a**) WC-6Co, (**b**) WC-8Co and (**c**) WC-12Co after undergoing electrochemical tests in 1 M H_2_SO_4_.

The SEM and optical micrographs for the WC-Co alloys are presented in Figure 8 and Figure 9, respectively. The SEM micrographs of WC-6Co and WC-8Co were all observed under the same magnification of 2000× except for WC-12Co, which was taken at 5000×. This was because the corrosion products left on the WC-12Co were very small compared to the other two WC-Co alloys.

The SEM micrographs of the three investigated WC-Co alloys displayed different topographical morphologies after electrochemical tests were conducted on them. However, WC-6Co and WC-8Co exhibited similar characteristics to each other that were very different from what was observed on the surface of the WC-12Co alloy. WC-6Co and WC-8Co both showed cracks on their surfaces where the corrosive medium penetrated. Sebeya [27] reported the same continuous cracked film on the surface of the WC-10Co sample he investigated in a similar corrosive environment. With WC-6Co (Figure 8a), the cracks formed by the corrosion product can be seen forming around individual WC particles and cracking on the Co phase, whereas with WC-8Co, the cracks were formed around an agglomeration of WC particles. It is not clear as to why this was the case as both alloys exhibited a negligible difference in their CR values. WC-12Co contained translucent patches of the corrosion product and not the cracked layer seen with WC-6Co and WC-8Co. The corrosion product layers observed on the surface of the WC-12Co alloy were discontinuous and had different patch sizes and did not seem to have severely penetrated and attacked the binder as observed with the other two WC-Co alloys. The WC-12Co alloy’s resistance to severe corrosive attack can be attributed to its refined and densely packed WC structure, which prevents the Co phase to be excessively exposed to the corroding media [29,32]. The optical micrographs of the WC-Co alloys show WC-8Co (Figure 9b) as having the most severe corrosion attack on its surface. Localised corrosion in the form of large pits can be seen occurring mostly with the WC-8Co alloy, which was also observed to be the case in the work conducted by Bricín et al. [26].

#### 3.3.2. HCWCI Alloys

**Figure 10:**
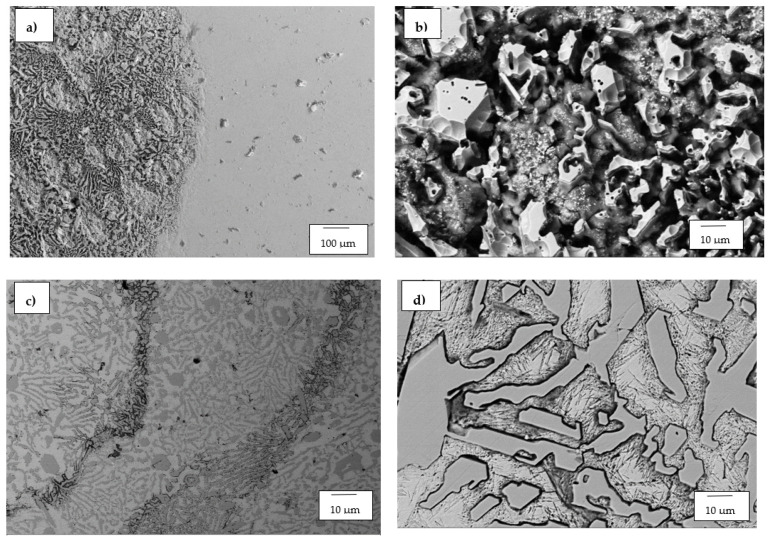

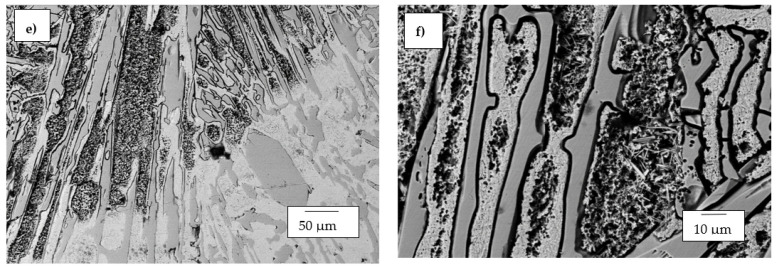
SEM micrographs of: (**a**,**b**) HCWCI-1, (**c**,**d**) HCWCI-2 and (**e**,**f**) HCWCI-3 after undergoing electrochemical tests in 1 M H_2_SO_4_.

The SEM for the HCWCI alloys are presented in Figure 10. Figure 10a–f present the SEM micrographs of HCWCI-1, HCWCI-2 and HCWCI-3, respectively.

It was observed that HCWCI-1 experienced the most severe corrosion attack after polarisation tests in 1 M H_2_SO_4_. The appearance of the corroded surface of the HCWCI-1 alloy correlated with its high current density and corrosion rate. HCWCI-1 underwent uniform corrosion damage on its surface, and this is characterised by the manner in which corrosive medium dissolved the matrix, leaving a slightly corroded skeleton structure of the primary eutectic carbides.

The corroded surface of the HCWCI-2 alloy (Figure 10c,d) portrays a strange corrosion morphology. Some regions of the surface (Figure 10a) that were completely exposed to the corrosive medium appear like they did not undergo any corrosive attack. However, upon close inspection, chemical attack was slightly visible on the matrix, proving that there was evidence of corrosion that took place. Additionally, the corroded grain boundaries around the Cr-rich primary carbides provide further proof that the surface did in fact undergo corrosion. Lack of obvious corrosion mechanisms like pitting or cracks in the matrix of HCWCI-2 is an indication that the alloy exhibited good corrosion resistance behaviour during the electrochemical tests. This was also confirmed by the same observation recorded by Beimeng et al. [54] and the alloy’s low current density and corrosion rate.

Similar to HCWCI-1, HCWCI-3 was expected to exhibit a severe corrosion attack on its matrix. This was expected because HCWCI-3 displayed a similar structure to that of HCWCI-1, which consists of a relatively higher volume fraction of eutectic primary carbides than HCWCI-2. HCWCI-1 and HCWCI-3 suffered more from localised corrosion than HCWCI-2 as shown in Figure 10. This phenomenon was also observed by El-Aziz et al. [36] where the structure that contained a volume fraction of eutectic primary carbides exhibited the most severe localised corrosion in the form of pits and microcrevices.

The only characteristic that the HCWCI alloys had in common was the inert behaviour the primary carbides exhibited during anodic polarisation tests. The corrosive damages in all the alloys were observed occurring in their matrices.

#### 3.3.3. The Hadfield Steel

**Figure 11:**
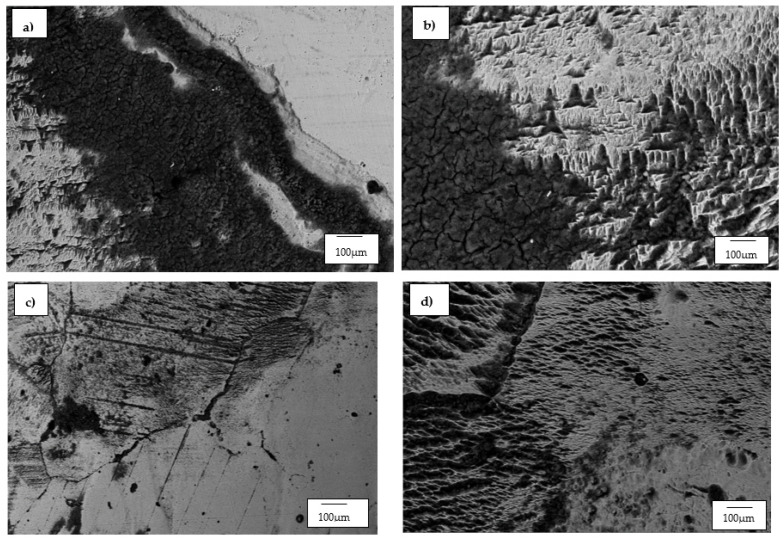
SEM micrographs of Hadfield steel after undergoing electrochemical tests in 1 M H_2_SO_4_ showing: (**a**) corrosion product on grain boundaries, (**b**) morphology after corrosion referred to as ‘Hills-and-valleys’, (**c**) corrosion product on slip-bands, and (**d**) corrosion product on austenite matrix.

The SEM micrographs for the Hadfield steel are presented in Figure 11. The micrographs focus on the corrosive mechanisms that occurred on the austenite grains, the carbide-rich grain boundaries and the carbide inclusions within the austenite grains.

The corrosive product (passive layer) is shown in Figure 11a,b characterised as a cracked dark-grey layer covering some parts of the corroded surface. The product layer is observed segregating within and around pits, grain boundaries and regions that are rich in carbide inclusions. The vertical black lines seen in Figure 11c shows that the twin boundaries also suffered chemical attack during the polarisation test. The same mechanisms were reported by Garfias-Garcia et al. [44] and Zhang and Zhu [45]. It is also noteworthy that there are large amounts of corrosion products that appeared as pits and craters after they were unintentionally delaminated during or after the electrochemical test. These large craters and micropores are shown more prominently in Figure 11c.

Figure 11b shows the occurrence of what is referred to as hills-and-valleys morphology observed on the corroded regions. The same type of morphology was reported by Ozgowicz et al. [42], and they reported that the observed surface after polarisation tests was a result of a mechanism known as the hydrogen depolarisation, which is common on surfaces that have undergone corrosion in an acidic medium.

## 4. Discussions

The electrochemical corrosion behaviour of the WC-Co cemented carbides, HCWCI alloys and the Hadfield steel was investigated by conducting electrochemical polarisation tests in 1 M sulfuric acid. It was observed that all of the investigated materials (except HCWCI-2) showed an active-psuedopassive transition behaviour during electrochemical tests. Figure 7 shows the potentiodynamic polarisation curves of the samples indicating the psuedopassivity region wherein maximum current densities were reached, which were then followed by a slight decrease. This feature was observed and outlined by Human and Exner [18], Marimuthu and Kannoorpatti [51] and Esmailzadeh et al. [55]. Moreover, from the obtained results, it is clear that the WC-Co alloys as a group behave similarly and have similar E_corr_ and i_corr_ values and therefore, similar corrosion rates. In the same fashion, the HCWCI behaved similarly and displayed closely related open circuit corrosion potentials and corrosion currents. There was hardly a difference between the corrosion current densities of all of these alloys, and they all show closely related corrosion rates.

The comparative behaviour of the WC-Co alloys in 1 M H_2_SO_4_ is shown in Table 4 and Figure 6 and Figure 7 WC-12Co showed better resistance to corrosion compared to the other two WC-CO alloys. This conclusion was characterised by the low current density and corrosion rate that WC-12Co exhibited after undergoing electrochemical tests.

**Figure 12:**
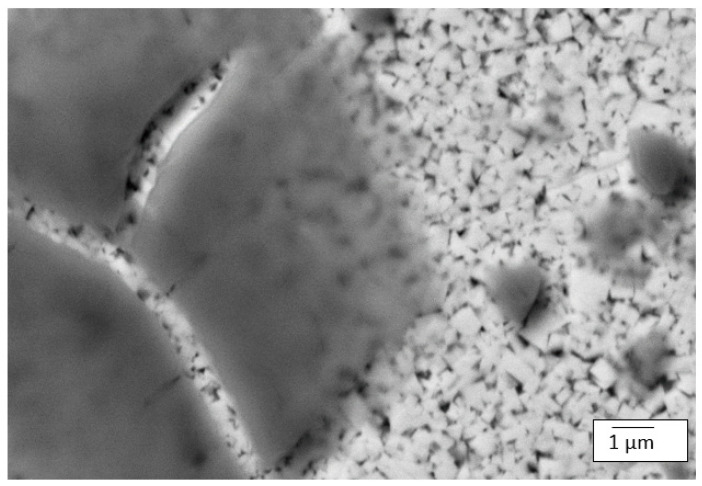
SEM micrograph of WC-12Co alloy showing the cracked translucent layer of corrosion product.

The SEM micrographs of the alloys that were taken after the alloys were exposed to the corrosive environment revealed that WC-6Co and WC-8Co experienced severe corrosion attack on their surfaces. The preferential manner in which the acidic medium selectively dissolved the grain boundaries around the WC particles lead to the same conclusion reported by Human and Exner [18], Sutthiruangwong and Mori [33], Thanjekwayo [56] Pugsley and Sockel [57], that the Co binder phase was attacked more vigorously compared to the WC grains. Figure 12 shows the SEM micrograph of WC-12Co alloy where the cracked translucent layer of the corrosion product can be seen. WC-12Co’s good resistance to corrosion can thus be attributed to the manner in which the WC particles were more exposed to the corrosive medium than the Co binder, hence behaving as a shield against the dissolution of the Co binder. So instead of the acidic medium attacking the alloy, it created a translucent corrosion product layer that covered the surface of the alloy, which could or could have not assisted as a protective shield against further corrosive attack.

**Figure 13:**
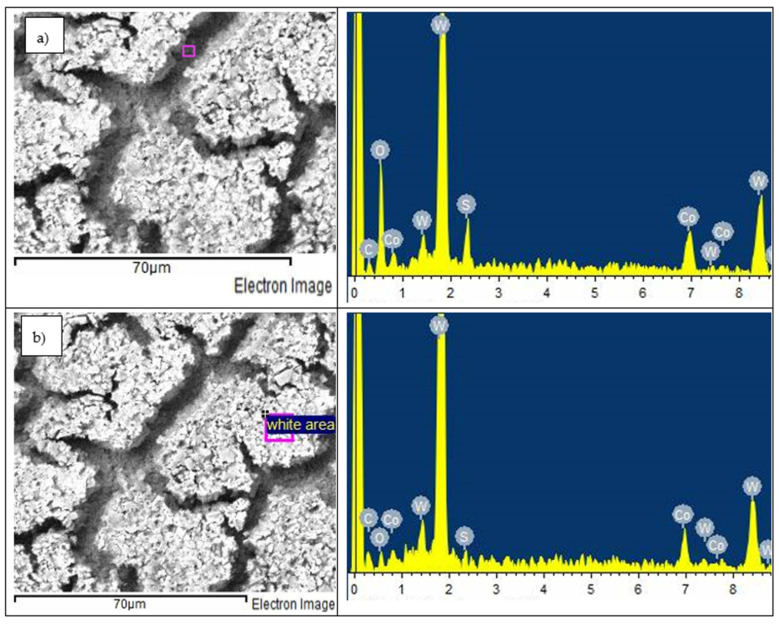
(**a**) SEM-EDS micrograph of WC-8Co showing (**b**) EDS analysis on the corrosion product layer after undergoing an electrochemical corrosion process in 1 M H_2_SO_4_.

**Figure 14:**
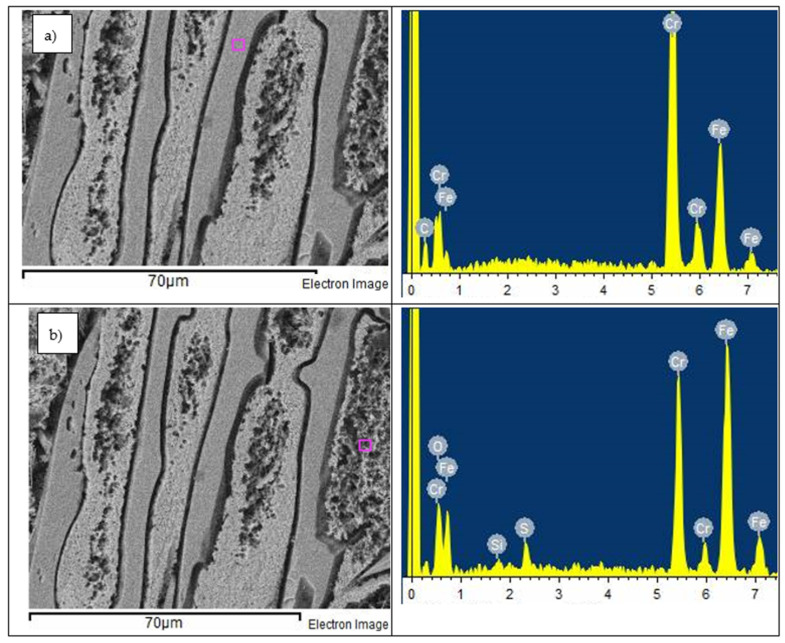
SEM-EDS micrograph of HCWCI-3 showing EDS analysis on: (**a**) primary carbide, and (**b**) needle-like austenitic matrix after undergoing electrochemical corrosion process in 1 M H_2_SO_4_.

**Figure 15:**
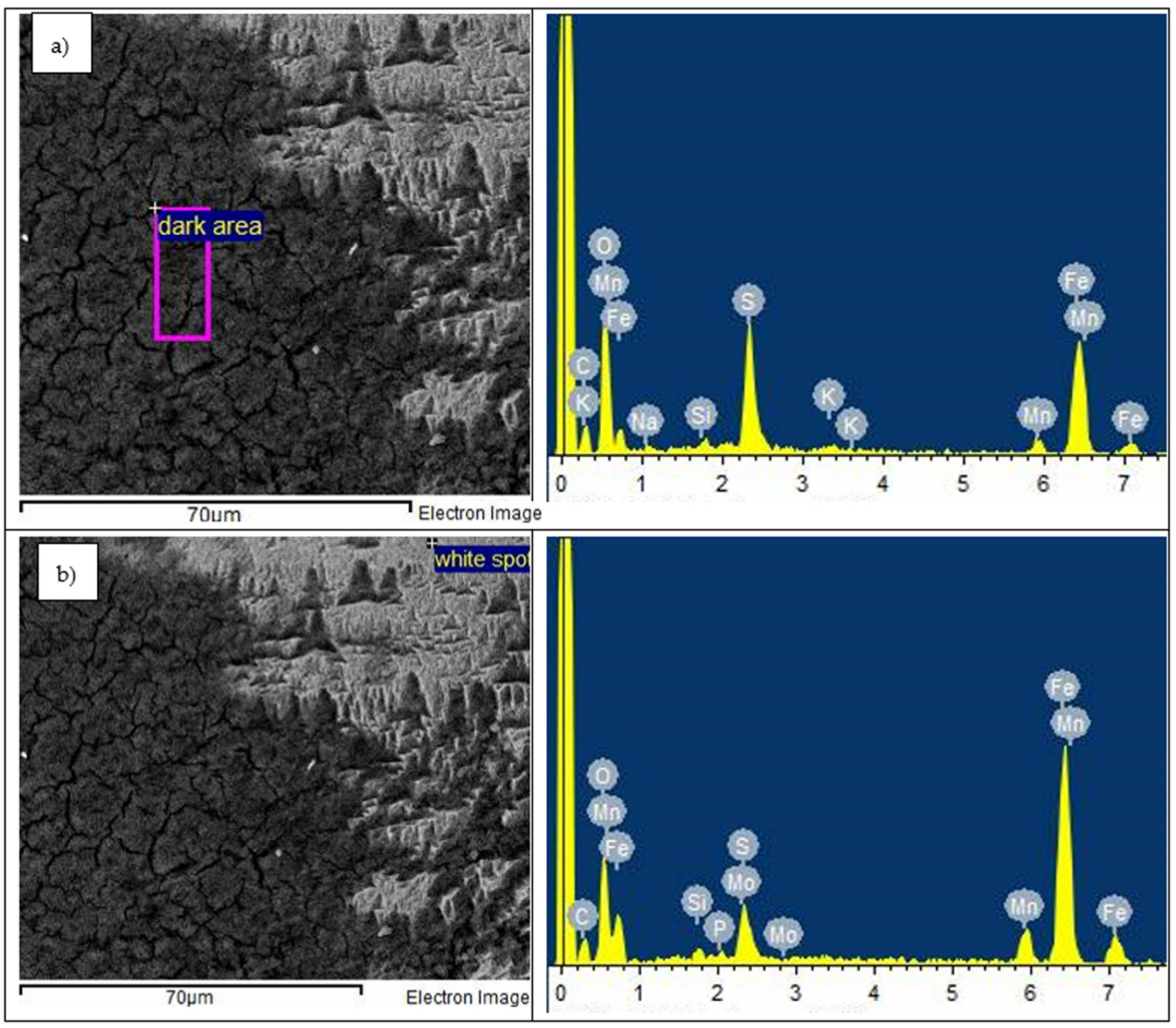
SEM-EDS micrograph of the Hadfield steel showing EDS analysis on: (**a**) darkened area of the corrosion product, and (**b**) lighter area of the corrosion product after undergoing electrochemical corrosion process in 1 M H_2_SO_4_.

The EDS results given in Figure 13, Figure 14 and Figure 15 are typical of the results obtained from the WC-Co, HCWCI, and Hadfield steel alloys respectively. EDS analysis of WC-8Co (Figure 13) showed the preferential dissolution of the cobalt binder. This was characterised by the decreased cobalt content and the traces of tungsten, carbon, sulphur and oxygen found in the cobalt phase. The EDS results also show very low traces of sulphur on the WC phases. This behaviour was also observed by the work conducted by Thanjekwayo [56] on the electrochemical work performed on WC-Co alloys with varying ruthenium contents.

Different contents of chromium and other alloying elements can significantly influence the corrosion behaviour of HCWCI alloys. However, only the effect of chromium, carbon and the morphology of the microstructure (matrix and the second phase) and the effect they had on the resistance of corrosion of HCWCI alloys will be discussed.

HCWCI-2 had the lowest value for corrosion current density and therefore, the lowest corrosion rate. This behaviour can be attributed to the alloy’s chromium and carbon content and the morphology of its occurring primary carbides. HCWCI-2 exhibited a better response to corrosion than HCWCI-1 because it contained a higher content of chromium [35]. Moreover, since carbon is the main carbide-forming alloying element in cast irons, less of it means there was excess chromium retained in the matrix, thus improving the overall resistance of the alloy. The same observation was recorded in work conducted by other researchers [34,36,58].

HCWCI-3 contained the highest chromium content of the three alloys, and it was thus expected that it would exhibit the best resistance to corrosion, but the opposite was observed. However, an increase in carbon was also observed, thus affecting the Cr/C ratio and reversing the effects of chromium. This ultimately had an adverse influence on the resistance of the alloy.

Figure 10a,b,e,f show that the alloys that contained high-volume fractions of primary eutectic carbides, HCWCI-1 and HCWCI-3, suffered severely from localised corrosion. El-Aziz [36] and Poolthong et al. [59] observed the same trends. They recorded that the matrix phase close to the eutectic carbides was deprived of chromium, causing the extensive corrosive attack seen with the two alloys. The black lines seen encapsulating the primary Cr-rich carbides in HCWCI-2 and HCWCI-3 are zones wherein the most depletion of Cr was observed, showing that corrosion attack in 1 M H_2_SO_4_ was initiated around the carbides.

The hard primary carbides occurring in HCWCI-2 and HCWCI-3 were observed to be inert to corrosion attack, and the eutectic primary carbides seen in HCWCI-1 exhibited negligible damage during the electrochemical tests. The EDS result from the HCWCI-3 alloy (Figure 14) shows that there were no traces of sulphur on the bulky primary carbides, whereas the needle-like structure on the matrix contained a high composition of sulphur. This indicates that the corrosion behaviour of white cast irons strongly depended on the composition and morphology of their matrix.

Table 4, which is the summary of the electrochemical parameters of the investigated materials, shows that the Hadfield steel exhibited the poorest resistance to corrosion in 1 M H_2_SO_4_. The steel displayed the highest i_corr_ and therefore, the highest corrosion rate. However, Hadfield steel showed a similar E_Corr_ value when compared with the E_Corr_ values exhibited by the HCWCI alloys. This shows that the alloys (HCWCI and the Hadfield steel) experienced the same tendency to corrode. This was expected since both material types consisted mainly of austenite in their structures.

The regions where the surface exhibited the hills-and-valley morphology was in fact tiny craters and pores where the surface suffered severe chemical attack. Other researchers that observed the same surface structure reported that this was a result of hydrogen penetrating deeper through the cracks of the passive layer (corrosion product), bringing about the rapid deterioration of the surface [42]. The hills-and-valley morphology is seen occurring predominately on the austenitic phase. This process is known as the hydrogen polarisation mechanism, and it occurs when the hydrogen ions in electrolytic form are reduced in cathodic areas of the metal by electrons to gaseous hydrogen [60]. This corrosion process caused causes tiny craters that are close together, separated by a network of strips of uncorroded metal that look like laths (Figure 11d). Figure 15, which is the EDS results of the Hadfield steel surface, show that these uncorroded laths did not contain sulphur,

It was reported by other researchers that the severely attacked surfaces were as a result of the protective passive layer being removed during the polarisation process, causing more damage to the exposed and unprotective surface [40,42,44].

The dark lines seen around the grain boundaries, metal-carbide inclusions and the non-metallic inclusion, such as sulphide inclusions, are regions that Garfias-Garcia et al. [44] reported were caused by hydrogen penetrating deeper into the surface of the steel. Abreu et al. [61] adds that areas such as grain boundaries, twin boundaries and non-metallic inclusions are regions where the accumulation of hydrogen is mostly seen during corrosion.

The three HCWCI materials considered in this study have all proven to perform exceptionally well in resisting wear, and from this work, the corrosion resisting properties of the HCWCI-2 alloy have been shown to be the best. However, from a practical viewpoint, the material to be used as protective lining plates for chutes and skips in the mining industry must be light and relatively inexpensive. The cemented carbide materials would probably resist wear and corrosion very well, but their weight would place extra stress on any structure, as they are generally double the density of mild steel. The Hadfield steels are the least inexpensive; however, these would need very high force of impacts to develop their work-hardening ability, which is not always guaranteed. The HCWCI alloys are a good option as these are approximately the same weight as structural steels used to construct chutes and skips. With the increasing operating costs in the mining industry in recent years, the costs of the HCWCI alloys (relative to the cemented carbide grades), as well as their ability to resist both wear and corrosion, would make them a good option for practical use.

## 5. Conclusions

The corrosion behaviour of different grades of WC-Co, high-Cr white cast irons and Hadfield steel in an acidic medium were investigated using electrochemical techniques. The main conclusions obtained from this study are as follows:

1. The WC-Co alloys behaved similarly as a group and displayed similar electrochemical trends. In the same fashion, the iron-based alloys (HCWCI and the Hadfield steel) showed similar behaviours. However, as a group the WC-Co alloys performed better and are more corrosion-resistant than the HCWCIs and the Hadfield steel.
2. The WC-12Co alloy, which contained the highest Co binder content and the finest WC particles in its structure, displayed the best resistance to corrosion compared to the other two WC-Co alloys. This characteristic can be attributed to its densely packed WC particles which shielded it against the aggressive dissolution of the binder. WC-12Co also contained traces of Cr in the form Cr_3_C_2_, which further contributed to the alloy’s resistance to corrosion. Microstructure is hence an important characteristic to consider in terms of the corrosion resistance of the alloys from a specific group, not only chemical composition.
3. The corrosion resistance of the HCWCI group was shown to be strongly influenced by the volume fraction of Cr-rich primary carbides. HCWCI-2 (22 wt% Cr), which consisted of larger rod-like primary carbides in its structure, displayed exceptional resistance against corrosive attack. Although not as good as the WC-Co alloys, this level of corrosion performance indicates that the experimental HCWCI alloys can be developed into cost-effective alternatives for the mining industry.
4. HCWCI-2 recorded a corrosion resistance that was superior to all the investigated specimens. While one clearly would have to consider potential wear conditions as well, its surface succumbed to the least corrosive degradation in comparison to the other investigated alloys in this study. The Hadfield steel exhibited the poorest resistance to the corrosive attack and suffered the harshest degradation to its surface. Therefore, although it is widely perceived to be a suitable material for mining applications where both wear and corrosion must be taken into consideration, it should in the opinion of the authors not be recommended for use.
5. Based on the work conducted in this study and the results thereof, the most eligible corrosion resisting material for the proposed application of serving as a protective liner for chutes and skips in acidic environments is HCWCI with a Cr content of 22 wt%.
6. One should be cautious to not only be guided by corrosion resistance of the evaluated materials for application in chutes and liners for the mining industry, as wear resistance is also of great importance in such environments. A follow-up study is therefore in progress on the wear behaviour of the evaluated alloys to arrive at a final decision of whether the more expensive WC-Co alloys can be replaced by a new group of HCWCI alloys.

## Figures and Tables

**Table 1:** Nominal composition of the investigated WC-Co alloys.

Samples	Composition (wt%)			
	W	C	Co	Cr
WC-6Co	86.0	7.9	6.1	---
WC-8Co	83.1	9.1	7.8	---
WC-12Co	79.6	7.5	12.0	0.9

**Table 2:** Nominal compositions of the investigated HCWCI alloys and the Hadfield steel.

Sample	Composition (wt%)												
	Cr	Si	Fe	Mn	S	Ni	V	P	Mo	Cu	Al	N	C
HCWCI-1	20.2	0.2	60.0	0.7	---	---	0.5	---	---	---	---	---	Bal.
HCWCI-2	22.2	0.4	65.2	---	---	---	---	---	---	---	---	---	Bal.
HCWCI-3	24.5	0.9	58.2	0.9	0.3	1.3	---	---	---	---	---	---	Bal.
Hadfield steel	0.34	0.58	85.0	12.4	<0.001	0.04	---	0.035	0.46	0.03	0.03	0.02	Bal.

**Table 3:** Summary of the primary carbide area range of the HCWCI alloys, WC grain sizes and the austenite grain sizes of the Hadfield steel.

HCWCI Alloys (µm^2^)			WC-Co Alloys (µm)			Hadfield Steel (µm)
HCWCI-1	HCWCI-2	HCWCI-3	WC-6Co	WC-8Co	WC-12Co	HS
25–1450	280–5330	65–2745	2.2	2.3	0.4	300

**Table 4:** Electrochemical parameters of the investigated materials.

Sample	E_corr_ (V)	i_corr_ (µA/cm^2^)	CR (mmpy)
WC-6Co	−0.189 ± 0.015	399.2 ± 1.2	0.09 ± 0.01
WC-8Co	−0.199 ± 0.01	359.2 ± 1.1	0.10 ± 0.02
WC-12Co	−0.149 ± 0.02	185.5 ± 2.4	0.04 ± 0.06
HCWCI-1	−0.423 ± 0.033	1033.4 ± 14.2	0.35 ± 0.01
HCWCI-2	−0.401 ± 0.014	9.96 ± 1.7	1.93 × 10^−3^ ± 0.58
HCWCI-3	−0.437 ± 0.021	801.2 ± 2.3	0.19 ± 0.2
HS	−0.406 ± 0.001	1084.7 ± 10.8	0.45 ± 0.17

## Data Availability

Not applicable.
